# A Practical Treatise on Materia Medica and Therapeutics, with Especial Reference to the Clinical Application of Drugs

**Published:** 1907-04

**Authors:** 


**A Practical Treatise on Materia Medica and Therapeutics,** with especial reference to the clinical application of drugs. By **John V. Shoemaker,** M.D., LL.D., Professor of Materia Medica, Pharmacology, Therapeutics, and Clinical Professor of Diseases of the Skin in the Medico-Chirurgical College of Philadelphia. Sixth edition. Octavo, 1244 pages. Philadelphia: F. A. Davis Company. (Price, Cloth, $5.00; sheep, $6.00, net prices).

Here is a sixth edition which carries its license for publication in the new and necessary material contained within its covers.

Always a valuable and dependable work, it has now been brought up to the very highest type of efficiency. The alterations in pharmacal nomenclature, the changes in strengths of various preparations and new titles which have been given ordinarily useful drugs, have made this revision almost a stern necessity, and the book in every respect now corresponds to the United States and British pharmacopeias. New therapeutic agents are given the fullest consideration warrantable according to their importance, among them being included the Roentgen ray, the Finsen light, serum therapy, animal extracts, vibrotherapy and hydrotherapy in all their newest phases. The book has been increased over 100 pages and is now a completed work.

